# The MALDI Method to Analyze the Lipid Profile, Including Cholesterol, Triglycerides and Other Lipids

**DOI:** 10.3390/cimb48010059

**Published:** 2026-01-02

**Authors:** David Aebisher, Izabela Rudy, Kacper Rogóż, Dorota Bartusik-Aebisher

**Affiliations:** 1 Department of Photomedicine and Physical Chemistry, Faculty of Medicine, Collegium Medicum, University of Rzeszów, 35-959 Rzeszów, Poland; 2 Student Scientific Club of Biochemists URCell, Faculty of Medicine, Collegium Medicum, University of Rzeszów, 35-959 Rzeszów, Polandkr117626@stud.ur.edu.pl (K.R.); 3 Department of Biochemistry and General Chemistry, Faculty of Medicine, Collegium Medicum, University of Rzeszów, 35-959 Rzeszów, Poland; dbartusikaebisher@ur.edu.pl

**Keywords:** MALDI, lipids, cholesterol, triglycerides, mass spectrometry, MALDI-MS, MALDI-MSI

## Abstract

Lipid profiling is a key element of modern biology and medicine, providing information on the structure, function, and dynamics of lipid metabolism in health and disease. This review presents the latest state of the art in the application of matrix-assisted laser ionization mass spectrometry (MALDI-MS) in lipidomics, with a particular focus on the analysis of cholesterol, triglycerides (TAGs), and the main classes of phospholipids and sphingolipids. The theoretical basis of the technique is discussed, including ionization mechanisms, matrix selection and mass analyzer configurations, and the influence of experimental parameters on spectral quality. The specificity of sterol and TAG ionization, challenges associated with ion suppression, and strategies for improving detection sensitivity and selectivity are discussed. Particular attention is paid to molecular imaging (MALDI-MSI), which enables spatial mapping of lipids in tissues and is of increasing importance in neurobiological, oncological, and metabolic research. The review highlights the advantages of MALDI for rapid lipid profiling and tissue analysis, while also pointing out technical limitations (e.g., difficulties in detecting sterols, matrix interference, limited quantification) and the need for method standardization. MALDI-MS appears to be a complementary technique to LC–MS/ESI-MS and DESI-MS, with great translational potential, particularly in the context of diagnostics, biomarker studies, and in situ lipid imaging.

## 1. Introduction

### 1.1. Definition of the Lipid Profile and Its Importance in Biology and Medicine

A lipid profile (lipidome) is a comprehensive characterization of all lipid species found in a given biological system, encompassing the identification and quantification of individual lipid molecules and their isomers and chemical forms. Lipids play key structural (membrane components), energetic (energy storage), and signaling (regulatory cascades) roles. Therefore, lipid profile analysis allows for the study of the mechanisms of metabolic, neurodegenerative, inflammatory, and cancer diseases, as well as the identification of potential biomarkers and therapeutic targets [1].

### 1.2. Basic Lipid Classes: Sterols, Triglycerides, Phospholipids, Sphingolipids, Free Fatty Acids

In lipidomics, several main lipid classes are distinguished: (i) sterols (e.g., cholesterol and cholesterol esters), responsible for membrane fluidity and precursors of steroid hormones; (ii) triglycerides (TAGs)—the main form of energy storage; (iii) phospholipids (glycerophospholipids, some sphingophospholipids)—essential components of the lipid bilayer and sources of lipid signals; (iv) sphingolipids (ceramides, sphingomyelins)—involved in cell signaling and apoptosis; and (v) free fatty acids (FFAs), which play energetic and signaling roles. Each of these classes includes numerous subspecies (various chain lengths and degrees of chain saturation)—significantly increasing the complexity of lipid analysis [2].

### 1.3. Traditional Lipid Analysis Methods (LC–MS, GC–MS, Enzymatic Assays)—Limitations

Traditionally, lipidomics utilizes chromatographic techniques coupled to mass spectrometry (LC–MS, GC–MS) and enzymatic assays to determine selected fractions (e.g., cholesterol or triglyceride concentrations) (Table 1). LC–MS/ESI-MS offers high sensitivity and molecular resolution, while GC–MS is widely used to determine fatty acids after derivatization. However, these methods have limitations: GC typically requires derivatization and applies only to volatile/thermally stable derivatives; LC–MS can be subject to ion suppression and requires time-consuming separation steps; and enzymatic assays provide only summary values for lipid classes, without information on specific molecular species. Furthermore, standardization and interlaboratory comparability remain a challenge in the clinical application of lipidomics [3,4].

### 1.4. The Growing Importance of MALDI-MS in Lipidomics

Matrix-Assisted Laser Desorption/Ionization Mass Spectrometry (MALDI-MS) has gained importance in lipidomics due to its rapid analysis of a wide range of lipid species (both polar and apolar), its tolerance to sample matrix contamination, and the ability to obtain spectra without excessive fragmentation. MALDI enables direct analysis of tissue extracts and preparations, and in its imaging variant (MALDI-MSI), spatial mapping of lipid distribution in tissue sections, which is particularly valuable in pathological studies (e.g., cancer, neurodegenerative diseases, metabolic dysfunction). Recent technical advances (matrix optimization, MALDI-2, hybrid arrays) have significantly improved the sensitivity and range of detectable lipids, including triglycerides and cholesteryl esters, which are not always easily analyzed using other techniques [12,13,14,15].

### 1.5. Purpose and Scope of the Review

The purpose of this review is to (i) provide a synthetic overview of the role of MALDI-MS in lipid profiling, with a focus on the detection of cholesterol, triglycerides, and other key lipid classes; (ii) discuss the advantages and limitations of MALDI compared to LC–MS/GC–MS; (iii) review recent methodological advances (matrices, sample loading techniques, MALDI-MSI, MALDI-2); and (iv) present perspectives on the translational and clinical applications of MALDI-lipidomics. This review integrates evidence from basic, methodological, and clinical studies, focusing on materials indexed in PubMed and NCBI resources.

### 1.6. Literature Search Methodology

This review is based on a comprehensive search of the PubMed/MEDLINE, Scopus, and Google Scholar databases. The search strategy utilized a combination of the following keywords: “MALDI-MS”, “lipidomics”, “MALDI imaging”, “cholesterol”, “triglycerides”, “phospholipids”, and “tissue preparation”. The literature review prioritized articles published within the last 15 years (2010–2025) to reflect the most recent technological advancements, although seminal historical papers describing the fundamental principles of MALDI were also included. Only peer-reviewed articles published in English were considered.

## 2. Theoretical Foundations of MALDI-MS

### 2.1. History and Development of the MALDI

Method MALDI (Matrix-Assisted Laser Desorption/Ionization) was developed in the 1980s as a “soft” ionization technique enabling the analysis of large, easily fragmented molecules (proteins, peptides, polysaccharides) without significant fragmentation. The key work of Karas and Hillenkamp established the concept of using a weak absorbent (matrix) coupled with a laser to desorption and ionize the analyte; a separate approach (Tanaka) [16,17] also contributed to the development of this field and was recognized with the Nobel Prize in 2002. In the following decades, MALDI expanded to include imaging of molecule distribution in tissues (MALDI-IMS), lipid analysis, and integration with various mass analyzers [16,17].

### 2.2. Ionization Mechanism in MALDI

**Figure 1:**
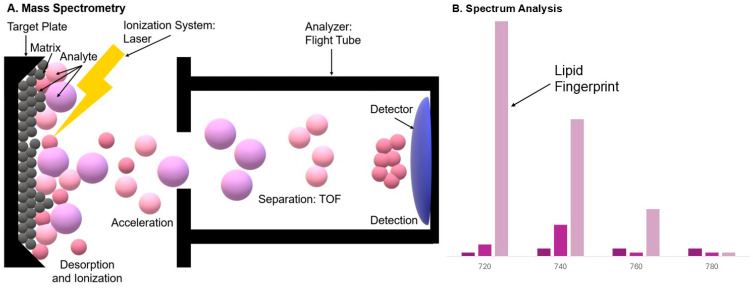
Mechanism of MALDI Ionization and Lipid Analysis. The figure illustrates the process of lipid analysis using Matrix-Assisted Laser Desorption/Ionization Mass Spectrometry (MALDI-MS). Panel (**A**) (Mass Spectrometry): The analyte (Lipid) is co-crystallized with the matrix. A laser pulse triggers desorption of the material and soft ionization, leading to the formation of singly charged ions. The ions are then separated in a mass analyzer (e.g., TOF) according to their mass-to-charge ratio (*m*/*z*). Panel (**B**) (Spectrum Analysis): The resulting spectrum provides a unique lipid fingerprint, enabling the identification of individual species such as phosphatidylcholine, triglycerides and ceramides.

In MALDI, the analyte is co-crystallized with an excess of a low-molecular-weight matrix, which strongly absorbs laser energy. Upon impact with a laser pulse, the matrix absorbs photons, rapidly desorbing the matrix layer along with the analyte. During rapid energy transfer and proton interactions, some of the analyte molecules ionize—usually forming ions of simple charges (most often single-voltage). The precise mechanism is complex and includes photophysical (absorption, superheating, sublimation), chemical (proton transfer, oxidation/reduction), and microthermal/kinetic components dependent on the matrix crystal morphology. In practice, ionization efficiency depends on matrix properties, laser wavelength, pulse energy, and the environment (e.g., acidic/base additives) [18,19]. Figure 1 shows the MALDI mechanism and a diagram of an example analysis result.

### 2.3. Most Commonly Used Matrices in Lipid Analysis

For lipid analysis in MALDI, a set of matrices is used, selected based on lipid type (polar/nonpolar) and ionization mode (positive/negative): 2,5-dihydroxybenzoate (DHB) is popular in positive mode and for lipid imaging due to its good solubility and fine crystal formation; CHCA (α-cyano-4-hydroxycinnamic acid) is effective for smaller lipids and peptides; norharmane demonstrates suitability as a matrix with a wide polarity range and allows for the detection of lipids in both polarities; 9-aminoacridine (9-AA) is preferred for the detection of lipids in negative mode (e.g., phospholipids, fatty acids) due to its favorable electron/proton transfer. Hybrid protocols and deposition optimizations (spray, sublimation, inkjet) also appear in the literature, significantly impacting signal yield and imaging resolution [20,21,22,23].

### 2.4. MALDI-Compatible Mass Analyzers

Traditionally, MALDI has most often been combined with a time-of-flight (TOF) analyzer due to the simplicity of the single-voltage ion source and wide mass range; TOF/TOF configurations additionally enable high-throughput fragmentation (MS/MS) in the second stage. In recent years, hybrid systems combining a MALDI source with high-resolution and mass-accurate analyzers, such as Orbitrap and Fourier Transform Ion Cyclotron Resonance (FT-ICR), have been developed, improving isobar identification and detailed lipid analysis (e.g., determining unsaturation and bond positions). Each analyzer has a trade-off: TOF offers speed and mass range, FT-ICR and Orbitrap offer higher resolution and accuracy at the expense of instrumental complexity and acquisition time [24,25,26,27].

### 2.5. Influence of Selected Experimental Parameters on the Quality of Lipid Spectra

The quality of lipid spectra in MALDI depends on many parameters: the choice and method of matrix application (sublimation vs. spray vs. spot deposition) influences crystal morphology and signal uniformity; the laser pulse energy and frequency determine desorption efficiency and fragmentation degree; spatial resolution in imaging depends on the laser spot size and matrix deposition method; chemical additives (e.g., salts, acids, bases) or the use of lipid-specific matrices (e.g., 9-AA for negatives) improve ionization selectivity and sensitivity. Furthermore, the use of high-resolution analyzers (FT-ICR, Orbitrap) and optimized acquisition conditions (number of scans, summation, calibration) significantly improve isobar identification and mass measurement accuracy. Comparative studies have also shown that different matrices and application protocols produce different lipid profiles, which requires careful validation of methods for a given sample type [22,28,29,30].

Consequently, defining a single set of experimental conditions for “general lipid profiling” involves a trade-off. Since no single matrix or polarity covers the entire lipidome with equal efficiency, the experimental condition of choice for broad coverage is typically a dual-polarity approach. Protocols often utilize a versatile matrix capable of ionizing in both modes (e.g., norharmane or DHB) or involve sequential acquisitions (positive mode for PC, TAGs, and SM; negative mode for PI, PS, PE, and FFAs). For unbiased general profiling, avoiding salt additives that selectively enhance specific adducts is also recommended, unless a specific class is targeted [23].

## 3. MALDI in the Analysis of Specific Lipid Classes

### 3.1. Analysis of Sterols, Including Cholesterol

Sterols (including cholesterol) are inherently nearly neutral and weakly polar molecules, which makes their direct ionization under typical MALDI conditions difficult. In practice, several mechanisms are observed leading to the appearance of sterol ions: (i) the formation of adduct ions with metal ions (Na^+^, K^+^) or proton ions when acidic additives are present; (ii) the formation of daughter ions by reaction with the matrix (reactive matrices that form covalent or semicovalent bonds/derivatives with the hydroxyl groups of sterols); and (iii) improving ionization efficiency through assistive techniques such as laser post-ionization (MALDI-2) or the use of mobile additives (anions, salts). Due to these mechanisms, the interpretation of sterol spectra requires consideration of the possibility of adducts and derivatives [31,32]. Because free cholesterol typically produces a weak signal in classical matrices (e.g., DHB, CHCA), the literature describes approaches based on: (a) reactive matrices (which chemically modify the sterol hydroxyl, improving its polarity and ion stability), (b) matrices with high charge transfer capacity (e.g., norharmane, which demonstrates good detection of nonpolar lipids), and (c) matrix application protocols and additives (e.g., metal salts or basic reagents) tailored to the formation of specific adducts. Furthermore, reports show that combining appropriate matrices with post-ionization techniques significantly increases the efficiency of sterol detection [13,21,31]. A growing number of studies and reviews demonstrate imaging and quantitative detection of cholesterol in tissues using MALDI-MSI: some reports use reactive matrices that convert cholesterol to a more ionizable form, others utilize tissue preparation procedures and/or laser post-ionization to enhance the signal. Brain and clinical tissue imaging studies have demonstrated the ability to map sterol distributions with subregional resolution, although quantitative accuracy and limitations due to adducts/sample preparation methods are still active research topics. In summary, MALDI offers real possibilities for cholesterol mapping but requires specific optimization of the matrix, acquisition conditions, and control of the derivatization reaction [31,32,33].

### 3.2. Triglyceride (TAG) Analysis

Triglycerides (TAG) are nonpolar lipids with relatively large masses and weak polarity. In MALDI, they are usually visible as adduct ions (with Na^+^, K^+^, or NH_4_^+^ ions in ammonia doping). In practice, TAGs often produce well-defined peaks in positive spectra, but their intensity and visibility depend strongly on the matrix, metal ion additives, and the presence of competing lipid classes (e.g., phospholipids) that may dominate ionization. Furthermore, in spatial imaging analyses, TAG detection requires a matrix/parameters that allow for efficient desorption of nonpolar molecules without excessive fragmentation [34,35]. One of the main challenges in TAG measurements is the ion suppression effect: the presence of strongly ionizing phospholipids (especially PC—phosphatidylcholine) can effectively suppress TAG signals in complex samples. Strategies described in the literature that minimize this effect include: (i) physical separation of lipid classes before analysis (e.g., fast solid-phase SPE, simple fractionation), (ii) selective chemical modifications (e.g., selective esterifications/derivatizations), (iii) the use of TAG-optimized matrices and loading protocols (or alternative LDI/NAPA platforms), and (iv) the use of ionic additives (e.g., NH_4_^+^) that promote the formation of the desired adduct and increase the TAG intensity relative to competitors. Comparative work shows that simple fractionation systems or matrix selection significantly improve the relative TAG detection [35,36,37]. Publications show several trends: (a) under classical MALDI-TOF conditions, it is possible to identify TAG components in oils and tissues, but the quantitative accuracy is limited by suppression and variable matrix effects; (b) the use of pre-MALDI separation (SPE, thin-layer chromatography) improves TAG detectability and quantification; (c) alternative LDI approaches (e.g., nanostructured surfaces—NAPA) or the use of matrices and protocols optimized for nonpolar lipids can outperform classical MALDI in TAG detection; and (d) in spatial imaging studies, TAGs are detected but require refined tissue preparation and acquisition conditions to minimize artifacts and suppression effects. In general, the literature indicates that MALDI is useful for TAG analysis, but often the best results are obtained when combining preparation (fractionation/purification) with good optimization of the matrix and ion additives [22,35,36].

### 3.3. Phospholipids

MALDI-MS demonstrates good ability to detect and distinguish phospholipid classes (e.g., PC, PE, PS, PI, PG) at the molecular weight level and adducts (Na^+^, K^+^, H^+^, NH_4_^+^). In practice, class differentiation is based on both mass differences (different polar heads and acyl chains) and characteristic adduct and fragmentation patterns obtained by MS/MS. However, species identification (e.g., double bond position, ester/ether isomerism) is sometimes limited by classical MALDI-TOF without additional separation or high-resolution MS/MS; isobaric and isomeric forms may require hybrid approaches (high resolution, MS/MS, derivatization) for unambiguous characterization. For tissue imaging, MALDI-MSI confirms phospholipid class distributions spatially, but structural resolution depends on the matrix and MS configuration used [38,39]. ESI-MS (usually coupled with LC) and MALDI-MS have complementary advantages and limitations in phospholipid analysis. ESI-LC-MS typically provides better isomer resolution, higher quantitative capabilities, and more developed pre-measurement separation protocols (which reduce ion suppression), making it preferred for detailed identification of lipid species and analysis of unsaturation or bond positions. MALDI-MS, on the other hand, offers rapid profiling and spatial imaging (MALDI-MSI), simpler preparation, and high tolerance for contamination, but often requires matrix optimization, ion additives, or additional techniques (FT-ICR, Orbitrap, MS/MS) to match ESI in structural resolution and quantification. In the literature, it is often recommended to combine both techniques: ESI-LC-MS for in-depth identification and quantification, MALDI-MSI for distribution mapping [21,38,40].

### 3.4. Sphingolipids

Sphingolipids (ceramides, sphingomyelins, glycosphingolipids) are detectable by MALDI, especially when using protocols optimized for these classes (matrix selection, additives, high-resolution analyzers). Ceramides and some sphingomyelins are relatively low in tissue abundance; therefore, studies using MALDI-FT-ICR or MALDI-Orbitrap and sensitivity-enhancing techniques (e.g., selective sample preparation, post-ionization) have demonstrated improved detection and enabled localization of these molecules in tissue preparations. Additionally, MS/MS allows for structural confirmation (e.g., chain length, unsaturation) of ceramides. However, the analysis requires careful validation, as some sphingolipids produce similar adduct signals and can be confused without high-quality fragmentation [41,42]. MALDI-MS (MSI) has found widespread use in biomedical research on sphingolipids: cancer studies use ceramide and sphingomyelin profiles to classify tumor types, monitor metabolic changes, and identify prognostic biomarkers; in neuroscience, MALDI-MSI enables spatial mapping of ceramides and their derivatives (e.g., sphingosine-1-phosphate (S1P)) in the brain and models of neurodegenerative diseases, providing insight into local lipid changes associated with pathology. Examples include both translational studies (human tissues) and animal models, where associations between dynamic sphingolipid changes and disease states have been demonstrated. These applications typically utilize high-resolution analyzers and MS/MS procedures to confirm identified signals [41,43,44].

### 3.5. Other Lipid Classes

**Figure 2:**
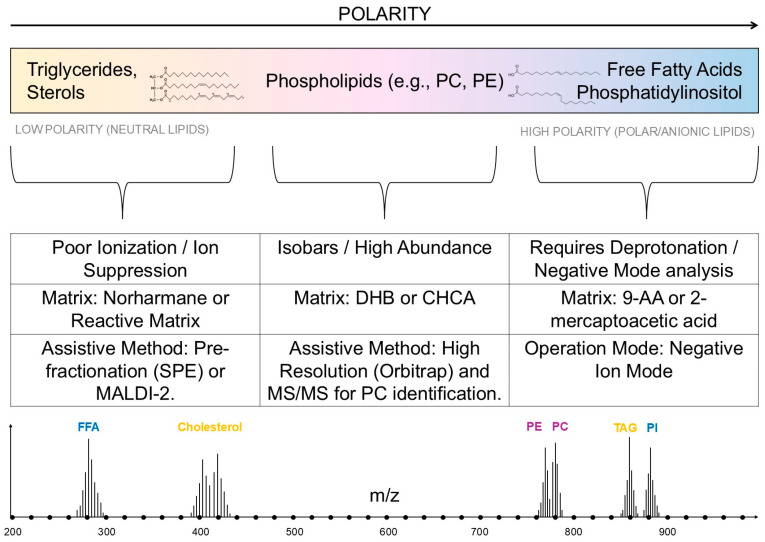
Optimization Scheme for MALDI-MS Lipid Analysis Based on Molecular Polarity. The diagram illustrates the necessity of adapting MALDI protocols to ensure the efficient detection of diverse lipid classes. The analysis is divided into three pathways based on polarity (from neutral to anionic lipids), which determines the methodological challenges: (1) Nonpolar Lipids (TAG, Sterols): Require dedicated matrices (e.g., Norharmane), ionic additives (NH4+), or assistive techniques (e.g., MALDI-2) to overcome poor ionization and ion suppression. (2) Glycerophospholipids (PC, PE): Ionize easily (high abundance), but full structural identification and isobar resolution necessitate advanced techniques (Orbitrap, MS/MS). (3) Anionic Lipids (FFA, PI): Require specific matrices (e.g., 9-AA) and operation in Negative Ion Mode for deprotonation and effective detection. The spectra at the bottom confirm that, after appropriate optimization, MALDI enables comprehensive lipid profiling across the entire polarity spectrum.

Detection of free fatty acids (FFA) by MALDI can be more challenging than by ESI due to their low polarity and strong ion competition. However, with the selection of appropriate matrices (e.g., 9-AA in negative mode or reactive matrices) and simple extraction procedures, FFA profiles can be analyzed directly from the preparations. However, for quantitative studies and detailed characterization of bond positions, ESI-LC-MS is usually preferred; MALDI, on the other hand, provides rapid spatial mapping and rapid profiling [21,39]. Lysophospholipids (LPC, LPE, etc.) are more polar than TAGs and often ionize well in positive mode in MALDI, enabling their detection in tissue samples and body fluids. However, like other lipid classes, their intensity can be modulated by the matrix and the presence of more strongly ionizing lipids; imaging analyses require optimization of matrix application and acquisition conditions to obtain reliable spatial distributions [39,45]. Glycerophospholipids (GPs), including PE, PC, PS, and PI, are among the most easily detected by MALDI and often constitute the dominant group in tissue spectra. MALDI-MSI directly depicts GP distributions in tissues, but full structural identification (e.g., distinguishing bond position isomers) requires MS/MS and/or hybrid techniques. In practice, good results are achieved by combining appropriate matrices, optimal tissue preparation, and high-resolution analyzers [21,38]. MALDI analysis of microbial lipids (especially MALDI-TOF) has gained popularity in clinical microbiology (species profiling) and metabolomics studies. Bacterial lipids (e.g., lipopolysaccharide/LPS, lipid A, specific phospholipids and glycolipids) can be detected directly or after simple preparation procedures; however, accurate identification and characterization of structures (e.g., fatty acid chain modifications) often require complementation with separation techniques or MS/MS. Alternative approaches (ambient ionization, NAPA) and microorganism-specific protocols that increase sensitivity and specificity have also been reported in the literature [46,47,48]. The characteristics of MALDI analysis of various lipids are shown in Figure 2.

## 4. MALDI Imaging (MALDI-MSI) in Lipid Analysis

### 4.1. Principles of MALDI Lipid Imaging

MALDI-MSI involves stepwise (raster) scanning of a matrix-coated tissue cross-section with laser pulses and recording a mass spectrum for each position. This produces a map of the *m*/*z* signal distribution that can be assigned to specific lipids or their adducts. Spatial resolution depends on the laser spot size and the matrix deposition method (e.g., sublimation, spraying, inkjet) [49]. Effective lipid imaging requires optimization: selecting a matrix compatible with lipid classes (e.g., DHB, norharmane, 9-AA for negative mode), a deposition protocol ensuring a thin, uniform layer, and laser and acquisition settings (number of pulses/position, scan summation). For nonpolar lipids and sterols, reactive matrices or post-ionization techniques (MALDI-2) are often used to increase sensitivity [50,51].

### 4.2. Applications in Tissue Studies

MALDI-MSI is widely used for mapping brain lipids. By analyzing regional profiles of phospholipids, sphingolipids, and sterols, researchers have gained insight into local metabolic changes associated with development, neurodegeneration, and genetic disease models. Combining MSI with immunohistochemistry or multimodal methods allows for the correlation of lipid signals with cellular markers [32,44]. In liver and cancer studies, MALDI-MSI helps identify areas of altered lipid metabolomics (e.g., tumor vs. healthy tissue zones, infiltration margins), detecting biomarkers potentially associated with disease progression and response to therapy; such analyses have been used in metastasis models and clinical trials sampling tumor tissue [52,53].

### 4.3. Imaging Cholesterol and Phospholipids

Cholesterol and its derivatives are more difficult to detect directly with classical MALDI due to low polarity and matrix interference. However, work using reactive matrices (chemical derivatization on tissue) and methods such as MALDI-IM (ion mobility) or MALDI-2 has significantly improved sensitivity and enabled the mapping of endogenous cholesterol in tissues. For phospholipids (PC, PE, PI, etc.), MALDI-MSI routinely provides clear signals and demonstrates their spatial heterogeneity, although structural identification (positional isomers, unsaturation positions) often requires MS/MS and/or high-resolution analyzers [13,20,31].

### 4.4. Clinical and Preclinical Applications

In the preclinical setting, MALDI-MSI is used to study disease models (e.g., neurodegeneration, cancer, metabolic disorders), to map the distribution of drugs and their metabolites, and to study the tissue microenvironment. In the clinic, the technique has potential as a tool supporting cancer diagnostics (sample classification, detection of lesion boundaries), but transition to routine use requires standardized protocols and validation [50,52,54]. Several studies show promising results in the context of lipid biomarkers associated with therapy response and prognosis, but the lack of uniform standards for sample preparation, calibration, and data analysis still limits widespread clinical implementation. Integration of MALDI-MSI with other imaging modalities (histology, molecular imaging) and automation of bioinformatic analyses are key to translation [54,55].

### 4.5. Limitations and Challenges of Imaging Methods

The main limitations of MALDI-MSI in lipid analysis include: ion suppression (competition between lipid classes), interference from matrix ions in the low *m*/*z* range, limitations in quantitative measurement (lack of universal internal tissue standards), and difficulties in distinguishing structural isomers without advanced techniques (MS/MS, IMS, high resolution). Furthermore, sample preparation (maintaining lipophilic integrity, preventing lipid migration during matrix application) is critical and often the dominant source of variability [13,20,56]. New solutions, such as MALDI-2 (laser post-ionization), the use of reactive/nonmetallic nanosurface matrices (NAPA), integration with IMS (ion mobility), and hybrid systems with FT-ICR or Orbitrap analyzers, alleviate some of these limitations (increased sensitivity, better isobar separation), but also introduce instrumental complexity and the need for additional method validation. Standardization, multicenter validation, and access to standardized references will be crucial for the wider adoption of MALDI-MSI in lipid studies [49,51,55].

## 5. Comparison of MALDI-MS with Other Lipidomics Techniques

### 5.1. MALDI vs. ESI-MS

Similarly to peptide analyses [57], MALDI and ESI offer complementary strengths in lipid studies. ESI (usually combined with LC) provides excellent results in resolution and quantitative analysis (it separates isomers chromatographically well, allows for seamless integration with MS/MS, and is very popular in routine lipidomics protocols), while MALDI is distinguished by its rapid acquisition speed, simple sample preparation, and the ability to perform direct spatial imaging (MSI). In practice, ESI-LC-MS is preferable when the goal is full, quantitative characterization of lipid species and isomer differentiation, while MALDI-MS is excellent for rapid profiling, tissue analysis, and studies where the preservation of spatial information is a priority. The two techniques often complement each other: identifications with ESI-LC-MS are used to confirm signals observed with MALDI-MSI [3,5].

### 5.2. MALDI vs. Ambient and High-Throughput Technologies (DESI, MALDESI, AEMS)

DESI (desorption electrospray ionization) is an ambient method that operates without the need for matrix application, offering the advantage of minimal preparation and often sharper spatial images. Comparative studies show that DESI-MSI produces higher ion intensities for many lipid classes and sharper image contrasts compared to classical MALDI-MSI, although MALDI may be more advantageous for some classes (e.g., ceramides, neutral lipids) depending on the matrix and conditions. The choice between MALDI and DESI therefore depends on the priority—maximum sensitivity/image contrast (DESI) or a broad lipid spectrum and the ability to work with reactive matrices/post-ionization (MALDI). Integration of both techniques in the analytical sequence (e.g., DESI → MALDI → histology) is used to obtain complementary information [58,59,60].

Beyond DESI, emerging high-throughput technologies such as MALDESI (Matrix-Assisted Laser Desorption Electrospray Ionization) and AEMS (Acoustic Ejection Mass Spectrometry) are expanding the analytical toolkit. MALDESI couples laser desorption with ESI post-ionization, effectively bridging the gap between MALDI imaging and ESI quantification capabilities [61]. Meanwhile, AEMS allows for contactless, ultra-fast sample injection (up to several samples per second), offering a throughput speed that challenges traditional vacuum-based MALDI methods in large-scale lipidomic screening [62].

### 5.3. MALDI vs. LC–MS/GC–MS

LC-MS (usually ESI) and GC-MS (usually after chemical derivatization, for volatile or fatty acid derivatives) are separation techniques that provide high lipid coverage and good quantification. GC-MS is particularly useful for the analysis of condensed and volatile fractions (e.g., fatty acid profiling after methylation), while LC-MS enables the analysis of a broad spectrum of non-volatile lipids with isomer separation [63,64]. As often observed in proteomics workflows [65], MALDI-MS does not replace separation techniques if maximum structural resolution and quantification are the goal, but it offers unique spatial imaging capabilities and rapid profiling without separation. In practice, the best results for complete lipid characterization are obtained by combining methods: LC/GC-MS for detailed identification and quantification and MALDI-MS (MSI) for tissue distribution mapping [63,64].

### 5.4. Sensitivity, Selectivity, Repeatability

Sensitivity: MALDI-MS can offer very high sensitivity for specific lipids (especially when using the appropriate matrix, ion additives, and/or MALDI-2 post-ionization), but its value is strongly dependent on the lipid class and sample conditions; ESI-LC-MS typically provides more predictable sensitivity across the entire lipid spectrum [20,66]. Selectivity: ESI combined with chromatography offers selectivity resulting from pre-ionization separation; MALDI offers selectivity resulting from matrix selection and acquisition conditions, and MS/MS/IMS, but is more susceptible to ion suppression effects between lipid classes [3,46]. Repeatability: Repeatability in MALDI-MS can be lower than in well-calibrated LC-MS workflows due to variability in matrix application, crystal morphology, and local differences in the tissue preparation. However, standardization of matrix deposition (sublimation, automatic sprays), use of internal standards, and summation of multiple scans improve reproducibility, making MALDI useful for inter-sample comparisons, especially in imaging studies [66,67,68].

### 5.5. Advantages and Limitations of MALDI in the Context of Lipidomics

Advantages of MALDI: rapid data acquisition and low processing costs (especially for imaging), the ability to detect a wide range of lipids with appropriate matrix selection, unique spatial mapping capabilities (MALDI-MSI), and compatibility with high-resolution analyzers (Orbitrap, FT-ICR) and post-ionization techniques that enhance traceability [46,57]. Limitations of MALDI: susceptibility to ion suppression (interclass competition), matrix interferences in the low *m*/*z* range, difficulties in quantitative analysis without well-chosen internal standards and protocols, limited isomer discrimination without MS/MS, IMS, or separation, and variability resulting from matrix application and tissue preparation. For this reason, MALDI is best used as part of an integrated lipidomics workflow (complementing LC/GC-MS and ESI), especially where spatial information is important [5,56,57].

## 6. Factors Affecting the Reliability of MALDI Lipid Analysis

### 6.1. Ion Suppression and Artifacts

Ion suppression is one of the most important sources of error in MALDI lipid analysis. In complex samples, certain lipid classes (e.g., phosphatidylcholines, strongly ionizing compounds) can dominate the ionization process and suppress the signals of other species (e.g., TAGs, sterols). This effect depends on the sample composition, matrix used, application method, and adduct properties (Na^+^, K^+^, NH_4_^+^). In practice, suppression is minimized by using sample fractionation, matrix mixtures selectively promoting different lipid classes, ionic additives favoring the formation of desired adducts or post-ionization techniques (e.g., MALDI-2), but complete elimination of the effect requires careful method validation and often confirmation of the results by an independent technique [69,70,71].

### 6.2. Influence of Matrix and Sample Application Method

While the chemical choice of matrix is fundamental (as detailed in Section 2.3), the application method (sublimation, automatic sprays, inkjet, “wet-interface,” etc.) has a significant impact on crystal morphology, lipid extraction from tissue, the degree of analyte delocalization, and consequently, the sensitivity and spatial resolution of images. Sublimation typically produces finer crystals and less delocalization, while “wet” sprays are sometimes better for extracting and detecting certain lipid classes; hybrid protocols and automated application improve reproducibility. The choice of matrix also influences adduct profiles and the presence of background ions associated with the matrix itself—therefore, comparative experiments of matrices and application methods are crucial for optimizing the method for a specific lipid class [20,72,73].

### 6.3. Lipid Stability and Process-Based Degradation

Lipids are susceptible to oxidation, lipolysis, and isomerization during sample collection, storage, and preparation (thawing, contact with solvents, length of exposure to air), which can lead to artifacts (e.g., formation of oxidized phospholipid derivatives and fragments). Studies have shown that even cut tissues and sections can undergo time-dependent degradation. Therefore, rapid freezing, temperature and storage time control, minimizing the number of freeze–thaw cycles, using antioxidants where possible, and applying standardized procedures during matrix application and drying are critical. Validation of the stability of specific lipid species and time-course tests should precede quantitative or comparative analyses [74,75,76].

### 6.4. Methodological Validation and Reproducibility

For MALDI-MS (especially MALDI-MSI) results to be reliable, comprehensive validation is necessary: the use of internal standards (synthetic lipids/dase), assessment of the response line (linearity), inter- and intra-individual reproducibility, limit of detection/quantity (LOD/LOQ) tests, and comparisons between instruments and sites. Automated matrix application, standardized tissue preparation protocols, and reporting of acquisition parameters (laser energy, pulse count, mass calibration) significantly improve reproducibility. Bioinformatics tools that correctly account for adduct, fragmentation, and background corrections (e.g., adduct and fragment annotation packages) are equally important, increasing the reliability of lipid identification [20,77,78].

### 6.5. Standardization—The Biggest Challenge in MALDI Lipidomics

**Figure 3:**
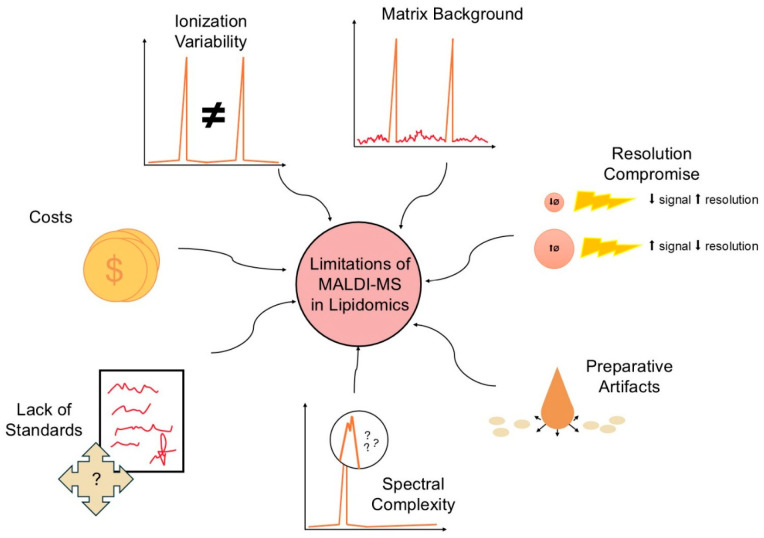
Major Methodological Limitations of MALDI-MS and MALDI-MSI in Lipidomics. The diagram summarizes key barriers limiting the technique’s quantification and standardization. Limitations include Ionization Variability (ion suppression, quantification error), Lack of Standards (universal reference materials, protocols), Spectral Complexity, Matrix Background, and MSI challenges: Resolution/Sensitivity Compromise and Preparative Artifacts (lipid migration).

Standardization of MALDI-MS and MALDI-MSI techniques in lipidomics remains the biggest challenge for translation into multicenter studies and clinical applications. Problems include the lack of universal tissue standards, variability in matrix deposition, differences in instrument configurations (different laser wavelengths, operating modes, analyzers), and the lack of uniform metrics for reporting image quality and quantitative results. Initiatives proposing reference protocols, interlaboratory benchmarking, and open databases of raw and processed data will be crucial. Therefore, mature clinical implementation of MALDI-MS requires standards for both experimental procedures and results reporting, as well as control sets for validation [20,77,79]. These and other challenges of MALDI techniques in lipidomics are presented in Figure 3.

## 7. Current Development Directions of MALDI Technology in Lipidomics

### 7.1. Matrix-Free Matrices and Laser Ionization in LDI

“Matrix-free” techniques (LDI without a classic organic matrix)—including DIOS (desorption/ionization on silicon), nanostructured surfaces (nanopores, gold/nanotube), and Surface-Assisted/Nanostructure-Assisted LDI (SALDI/NALDI) platforms—are gaining increasing interest because they remove typical matrix interferences in the low *m*/*z* range and improve the visibility of small and medium-sized molecules, including many lipids. Such approaches often provide a spectral background significantly cleaner than classic MALDI and can improve sensitivity to nonpolar compounds through surface effects (plasmonic, thermal) that promote desorption and charge transfer. The literature describes both silicon surfaces (DIOS) and nanostructured metals (e.g., nanostructured gold), as well as hybrid platforms—all of these solutions offer alternatives to organic matrices for the analysis of difficult-to-ionize lipids [80,81,82].

### 7.2. Nanomaterials and Hybrid MALDI Matrices

Another strong trend is nanomaterials used as an “active” component of matrices or as entirely new matrices: graphene/graphene-oxide, metallic nanoparticles (Ag, Au), metal oxides, Metal–Organic Frameworks/Covalent Organic Frameworks (MOFs/COFs), and quantum dots. Nanohybrids (e.g., graphene + organic matrix, nanosurfaces coated with functional layers) combine the advantages of selective adsorption of analytes with ionization enhancement. In practice, it has been shown that the use of nanomaterials can: (i) increase sensitivity for selected lipid classes, (ii) reduce matrix background at low *m*/*z*, (iii) enable selective adsorption of nonpolar lipids, which improves the signal-to-noise ratio in imaging. Reviews and experimental work document the rapid growth of nanosurface applications in lipidomics [83,84,85].

### 7.3. Integrating MALDI with Microfluidics

The integration of microfluidics with MALDI is a third approach: microfluidic systems (both flow chips and digital microfluidics/DMF) enable the automation of sample preparation, extraction, fractionation, or on-chip derivatization prior to deposition on the MALDI target. Such approaches offer specific benefits for lipidomics: controlled fractionation reduces ion suppression effects, automated deposition of small volumes improves reproducibility, and combining microfluidic droplets with precise deposition allows for the study of individual droplets/cells with high sensitivity. Studies also demonstrate that combining micropreparation with MALDI-MS can increase throughput and facilitate procedure standardization [86,87,88].

### 7.4. MALDI-MS for Rapid Clinical Diagnostics

MALDI-MS (especially MALDI-TOF configurations) has already established itself in microbiological diagnostics as a rapid, inexpensive, and efficient tool for microbial identification; work is currently underway to expand its clinical applications to include the direct detection and profiling of lipid biomarkers of disease (e.g., tumor markers, inflammation, metabolic changes) in tissue specimens and body fluid samples. In practice, rapid MALDI protocols enable the generation of lipid fingerprints in minutes to hours, raising the prospect of using the technique as a rapid screening test or to support clinical decisions, although rigorous clinical validation, standardized procedures, and regulatory approval are necessary [12,89,90].

### 7.5. Automation and High-Throughput Lipid Analysis

High-throughput MALDI-based platforms (HT-MALDI) combine automated sample preparation (96-well SPE, robotic matrix application, microfluidic preprocessing) with rapid spectrometer readout, enabling the simultaneous processing of hundreds to thousands of samples. This high-throughput capability is increasingly utilized for phenotypic screening applications. By capturing a comprehensive lipid “fingerprint,” MALDI-MS allows for the rapid classification of cellular phenotypes (e.g., monitoring cellular responses to pharmaceutical compounds or toxicological stressors) based on global spectral pattern changes rather than the absolute quantification of individual species. Such solutions are already used in the pharmaceutical industry and in screening studies (e.g., metabolism/drug resistance assessment, lipid profile monitoring)—automation reduces operational variance and increases reproducibility, but requires optimized extraction procedures, sample quality control, and integrated data analysis systems [91,92,93].

### 7.6. Combining MALDI with AI/Machine Learning in Spectral Interpretation

Growing MALDI-MS and MALDI-MSI datasets provide ideal conditions for the application of machine learning and deep learning (ML/AI) methods—from the classification of multidimensional lipid fingerprints (e.g., distinguishing cancer types), through automatic signal annotation and artifact removal, to the prediction of clinical outcomes. The literature describes both classical algorithms (SVM—Support Vector Machine, random forest) for pattern recognition in spectra and neural networks trained directly on MSI maps; ML also aids in the integration of data from other platforms (LC-MS, histology) and the creation of interpretable biomarker models. Key challenges include the need for large, well-labeled training sets, standardized pre-analysis processing, and rigorous model validation (cross-validation, external datasets) to avoid overfitting and ensure model portability between centers [94,95,96].

## 8. Clinical Applications of MALDI Lipid Analysis

### 8.1. Metabolic Diseases (Diabetes, NAFLD, Obesity)

Lipidomic studies using imaging techniques (MALDI-MSI) and profiling have demonstrated characteristic changes in the local and global content of specific lipid classes in metabolic diseases—for example, shifts in the profile of triacylglycerols, ceramides, and specific phospholipids in the liver in Non-Alcoholic Fatty Liver Disease/Non-Alcoholic Steatohepatitis (NAFLD/NASH), as well as indirect correlations between specific lipid species and insulin resistance and obesity. MALDI-MSI also enables the imaging of regional lipid distribution in tissue (e.g., periportal vs. pericoronary zones in the liver), which helps link metabolic molecular changes to morphology and pathology. Despite promising results, translation to routine diagnostics requires validation of biomarkers in blood samples and the establishment of quantification procedures [97,98].

### 8.2. Neurodegenerative Diseases (Alzheimer’s, Parkinson’s)

The brain has a unique, lipid-rich metabolism—alterations in gangliosides, sulfatides, ceramides, and selected phospholipids are consistently reported in models and samples of neurodegenerative diseases. MALDI-MSI (especially when combined with high-resolution analyzers and/or techniques such as MALDI-2) allows for mapping of these abnormalities regionally (hippocampus, cortex, white matter), identifying areas with altered lipid profiles associated with pathology (e.g., accumulation of oxidized lipids, decrease in certain sulfatides). Such spatial maps support hypotheses about the role of lipids in synaptic pathology, myelination, and neuroinflammation, but require further validation studies and correlation with clinical data [44,99].

### 8.3. Lipid Profile in Oncology

In oncology, MALDI-MSI is used to study tumor heterogeneity—lipid profiles differ between tumor zones, the microenvironment (e.g., areas of hypoxia, necrosis), and healthy tissue. Changes in phospholipid (PC, PE), ceramide, and glycolipid levels often correlate with invasiveness, malignancy grade, or response to therapy. MALDI-MSI helps identify local biomarkers that predict disease progression or indicate treatment-resistant zones, making it a promising technique for supporting surgery (mapping tumor boundaries) and translational research; however, widespread clinical use requires standardization and multicenter validation [54,100].

### 8.4. Applications in Cardiology (Dyslipidemia, Atherosclerosis)

Lipid analyses of vascular tissues and atherosclerotic plaques show that specific lipid species (e.g., oxidized phospholipids, cholesteryl esters, specific diacylglycerols) are associated with plaque instability and the risk of cardiovascular events. MALDI-MSI allows for spatial mapping of the distribution of these lipids within the plaque (lipid core, inflammatory zones, attachments). Such data can support studies of plaque destabilization mechanisms and the search for molecular markers of plaque instability. Combined with body fluid analyses and lipoprotein profiling, MS approaches offer the potential for more precise cardiovascular risk assessment, although clinical implementation requires standardized protocols and prognostic evidence [101,102].

### 8.5. Possibilities of Using MALDI as an In Vivo/Ex Vivo Diagnostic Tool

Currently, MALDI is an ex vivo technique (analysis of excised tissues, biopsies, and autopsy specimens). However, the development of rapid protocols (rapid preparation, automation, bulk acquisition) and integration with ML algorithms are paving the way for applications closer to clinical realities—for example, rapid, operational mapping of lesion boundaries during surgical procedures (ex vivo) or as part of the diagnostic workflow in pathology. Ambient technologies (DESI) and work on miniaturization and hybridization (e.g., MALDI-TOF at the point of care) increase the prospects for rapid screening tests, but current barriers include regulations, the need for clinical validation, and the logistics of integration into routine medical processes. In vivo applications (direct patient analysis) remain conceptual or experimental for now due to technical and safety constraints [78,103,104].

## 9. Limitations of the Method and Unresolved Problems

One of the main and often criticized limitations of MALDI-MS in lipidomics is the difficulty of achieving reliable, absolute quantification. Unlike techniques such as LC-MS, in MALDI the signal for a given lipid strongly depends on the ionization efficiency (which differs between lipid classes), matrix type, crystal morphology, and sample preparation conditions—which means that the peak intensity does not clearly reflect the analyte concentration in the sample [6,105]. One study combined separation (HPLC) with MALDI-TOF, allowing the quantification of lipid classes and species in lipoproteins—however, this approach requires additional steps (separation, fractionation), which reduces the advantages of MALDI’s simplicity. This limits the use of MALDI-MS as a quantitative method in routine lipidomics, especially when the goal is to compare concentrations between samples/patients [106]. MALDI-MS often exhibits large ionization variability between different lipid classes—difficult lipids (e.g., very nonpolar, neutral TAGs, sterols, some sphingolipids) may ionize very weakly or not at all, especially when more easily ionizable lipids (e.g., phospholipids with polar groups) dominate [105]. Furthermore, the presence of a matrix introduces background noise (matrix ions), especially in the low *m*/*z* range—which complicates the detection and analysis of small and medium-sized lipids. This limitation means that the lipid profile obtained by MALDI may be incomplete and biased towards more ionizable lipid classes, making it difficult to fully reflect the actual lipid composition [7,58]. Another barrier is the lack of universal internal standards and common protocols for MALDI-MS lipidomics. Due to the diversity of lipids (classes, polarity, adductivity, isobaric/isomerism) and the strong dependence of the signal on analytical conditions, it is difficult to develop a standard that works effectively for all lipids. Reviews indicate that matrix selection is often based on trial and error, which hinders interlaboratory reproducibility [7,105]. A critical limitation in detailed lipid profiling is the inability of standard MALDI-MS to differentiate lipid isomers. Lipids exhibit huge structural diversity through various forms of isomerism, including positional isomers (different locations of fatty acyl chains on the glycerol backbone, e.g., sn-1 vs. sn-2), double bond position isomers (e.g., Δ9 vs. Δ11), and geometric isomers (cis vs. trans). Since standard MALDI spectra rely principally on the mass-to-charge ratio (*m*/*z*) of intact ions, these isobaric species appear as a single peak. Consequently, without advanced fragmentation (MS/MS), ion mobility separation, or specific derivatization (e.g., Paternò–Büchi reaction), MALDI-MS typically yields only “sum composition” data (e.g., PC 34:1) rather than the precise molecular structure. This lack of specificity can obscure biologically significant changes in specific lipid subspecies [107,108,109]. Furthermore, the use of stable isotope-labeled standards for every lipid species—although theoretically a good idea for quantification—is practically impossible due to the complexity of lipidomes and the cost of synthesizing such standards. As a result, methodological validation (line of detection, dynamic range, reproducibility, accuracy) often remains limited and local; the lack of standards hinders data comparison between different laboratories or studies [4]. Three-dimensional lipid imaging (MALDI-MSI) faces several technical and conceptual challenges. First, achieving high spatial resolution (<10 µm, let alone subcellular) involves a compromise between laser spot size, signal sensitivity, and matrix crystal morphology—smaller spots typically yield weaker signals and require optimization of matrix application and acquisition. Second, lipid delocalization during sample preparation (solvent extraction, wet matrix application) can distort the true molecular distribution in the tissue, making biological interpretation difficult. Third, the spectral complexity of tissues—many isobars and adducting phenomena—necessitates the use of high mass resolution or MS/MS to correctly annotate signals; however, not every instrument allows for simultaneous high spatial resolution, high sensitivity, and fast throughput. Consequently, interpretation of MSI images requires careful experimental design, control of sample preparation processes, and often multimodal validation (e.g., correlation with histology, LC-MS) [53,55,110,111]. The lack of widely accepted, standardized protocols—including sectioning, storage, section preparation, matrix selection and application, laser parameters, and calibration and data annotation procedures—is one of the key factors limiting the comparability of MALDI-MSI results between laboratories. These differences impact reproducibility and hinder the development of interlaboratory databases. Specific procedures have been proposed in the literature (e.g., optimization of matrix deposition by sublimation vs. spray, serial washing protocols, and standardized acquisition parameters), but their adoption on a multicenter scale requires extensive comparative testing and community consensus. Translation into clinical trials will also require guidelines on quality control, internal tissue standards, and result reporting formats [104,112,113]. Equipment and operating costs constitute significant limitations. Modern MALDI-MSI platforms (especially those combined with Orbitrap or FT-ICR, small-spot lasers, automated matrix application devices, and MALDI-2/post-ionization systems) require a large capital investment, plus the costs of service, consumables, and personnel training. Furthermore, high spatial and mass resolution and comprehensive validation protocols extend measurement time, which translates into higher unit analysis costs. On the other hand, in selected applications (e.g., clinical microbiology), MALDI-TOF implementation has proven cost-effective in the long term, but the transfer of economic benefits to MALDI-MSI constellations in lipidomics is less obvious and requires evaluation in the context of the specific application (translational research vs. routine diagnostics). In practice, the choice of platform and investment scope must consider a trade-off between analytical capabilities and research budget and objectives [16,114,115].

## 10. Conclusions

MALDI-MS has gained the status of one of the key techniques in lipidomics: thanks to the ability to directly analyze tissues, extracts, and preparations without the need for chromatographic separation, and thanks to relatively quick and simple sample preparation, it allows for the acquisition of lipid profiles and—in the imaging version (MALDI-MSI)—maps of the spatial distribution of lipids in tissues (Table 2) [13,50]. Many studies have confirmed that MALDI-MS can detect a wide range of lipid classes—from phospholipids and sphingolipids to neutral lipids—although sensitivity depends on the matrix and analysis conditions. This makes MALDI-MS a valuable discovery tool in lipidomics, especially where spatial (tissue) structure and heterogeneity are crucial [53]. Compared to chromatographic techniques (e.g., LC-MS, GC-MS) and liquid-phase ionization (ESI-MS), MALDI-MS offers significant advantages: minimal preparation, the ability to analyze directly from tissue specimens, high throughput and measurement speed, and the unique ability to spatially image lipids (MSI), which provides insight into the location of molecules—not just their presence and quantity [22,90]. Furthermore, with a well-chosen matrix, MALDI can ionize lipids with a wide range of polarities and masses, making it a versatile tool for lipidomics studies [50]. Currently, the greatest developments and promise are related to: (a) improving matrices and nanomaterials—which enable better ionization of difficult lipids and reduce the spectral background; (b) integrating MALDI-MSI with high-resolution mass-resolution techniques (e.g., FT-MS/FT-ICR/Orbitrap analyzers) and with MS/MS and IMS techniques, which enables more detailed lipid identification (acyl chains, isomers, adducts); (c) developing automation and high-throughput analysis methods, which can facilitate scaling of studies; (d) combining spatial lipidomics (MALDI-MSI) with other “omics” (proteomics, metabolomics, transcriptomics), which will enable a comprehensive understanding of biochemical changes in diseases; and (e) applying bioinformatics tools (ML/AI) to analyze large spectral sets, which can accelerate biomarker identification and facilitate the classification of pathological conditions [21,53,116]. Although MALDI-MS and MALDI-MSI still face challenges (ionization, standardization, quantification), the potential of the technique as a diagnostic tool is significant. Thanks to its ability to map lipids in tissues, rapid profiling, and relatively low sample size, MALDI may prove useful in the diagnosis of metabolic, neurological, oncological, and cardiovascular diseases, particularly as a screening test or as a complement to histology and biochemical analyses. In the long run, the development of matrices, better analyzers, and standardized protocols, as well as integration with clinical and molecular data, may enable widespread use of MALDI-MS in personalized medicine and molecular diagnostics [50,90,117].

## Figures and Tables

**Table 1:** Comparison of lipid analysis methods: MALDI-MS vs. LC–MS vs. GC–MS.

Criterion	MALDI-MS [5,6,7]	LC–MS (ESI/APCI) [8,9]	GC–MS [10,11]
Required sample preparation	Minimal; often direct analysis or simple lipid extraction; requires matrix selection	Medium; requires extraction, sometimes purification, and chromatographic separation	High; requires extraction and mandatory derivatization (e.g., FAME)
Lipid range analyzed	Broad: phospholipids, sphingolipids, TAG, DAG, sterols; more difficult: FFA below low mass	Very broad, maximum versatility—virtually all lipid classes	Mainly FFA and sterols after derivatization; more difficult for phospholipids and sphingolipids
Sensitivity	High (matrix dependent); increases with MALDI-2	Very high	Very high after derivatization
Isomer resolution	Limited (no chromatographic separation)	High thanks to chromatography	High for volatile derivatives
Analysis speed	Very fast (seconds per spectrum)	Average (minutes to hours per analysis)	Average
Imaging capability (MSI)	YES—unique advantage (MALDI-MSI enables lipid mapping in tissues)	No (no integration with additional techniques)	No
Instrument complexity and operating cost	Average	High	Average
Resistance to matrix contamination	High tolerance to salt, urea and pollutants	Low to medium (ion suppression)	Low (requires pure extracts)
Clinical/translational utility	High in the context of rapid analysis and tissue imaging; rising in biomarkers	Very high—the gold standard of clinical lipidomics	Limited mainly to fatty acid analysis
Typical applications	Tissue lipidomics, tumor imaging, TAG and sterol analysis; rapid clinical analysis	Lipidome profiling, disease biomarkers, mechanistic studies	FFA, sterol analysis, food quality control, reference methods

**Table 2:** Advantages and limitations.

Categories	Advantages	Limitations	Sources
Lipid Analysis	Rapid and direct analysis of extracts and tissues; minimal preparation required	Quantification difficulties and different ionization efficiency between lipid classes	[118]
Multi-dimensional Imaging (MALDI-MSI)	Ability to map lipid localization in tissue; integration with histology	Lipid delocalization, limited spatial resolution with high sensitivity	[119]
Lipid Detection Range	Possibility of ionization of various lipid classes (phospholipids, sphingolipids, neutral lipids)	Weak ionization of some lipids (e.g., TAG, sterols)	[120]
Performance and Throughput	Fast measurements and high throughput; automation possible	High equipment and operating costs; requires advanced sample preparation	[121]
Development Potential	Integration with ML/AI and other omics; development of new matrices and nanomaterials	Lack of interlaboratory standards, limited reproducibility between studies	[122]
Clinical Applications	The perspective of personalized diagnostics; complementing histology	Requires further validation, standardization and integration with clinical data	[114]

## Data Availability

No new data were created or analyzed in this study.
